# Implementing TB control in a rural, resource-limited setting: the stop-TB Italia project in Senegal

**DOI:** 10.1186/s40248-018-0154-3

**Published:** 2018-11-09

**Authors:** Mama Moussa Diaw, Mamoudou Ndiaye, Niccolò Riccardi, Riccardo Ungaro, Riccardo Alagna, Daniela Maria Cirillo, Luigi Codecasa, Claudio Viscoli, Laura Ambra Nicolini, Giorgio Besozzi, on behalf of StopTB Italia Onlus

**Affiliations:** 1 Médecin coordonnateur lutte contre la TB, Région médicale de Thiès, Thiès, Sénégal; 2 District Sanitaire de Diofior/Département de Fatick, Diofior, Sénégal; 3 Infectious Diseases Clinic, Ospedale Policlinico San Martino, University of Genoa, Genoa, Italy; 4 TB Supranational Reference Laboratory, IRCCS San Raffaele Scientific Institute, Milan, Italy; 5 Regional TB Reference Centre, Villa Marelli Institute/ASST Niguarda Ca’ Granda, Milan, Italy; 6 Stop TB Italy Onlus, Milan, Italy; 7 Bureau Régional Immunisation et Surveillance Epidemiologique de Thiès, Avenue Malick SY prolongée BP 34A, Thiès, RP Sénégal

**Keywords:** Tuberculosis, Resource limited setting, Xpert MTB/RIF, Mobile chest-X-ray, Senegal

## Abstract

**Background:**

Since 2013 StopTB Italia Onlus supports the Senegalese National Tuberculosis Programme by improving diagnostic capability with technological interventions, ameliorating educational programs for health care personnel, rising awareness among civil society and providing economical support for patients during treatment. The purpose of our study was to assess the preliminary results of an interventional cooperation project in a peripheral health care facility in Senegal.

**Methods:**

An observational, retrospective, pre-post study was conducted to compare Tuberculosis (TB) retention in care and outcome between a one-year period before and a four-year period after.

**Results:**

Overall, 239 patients with active TB were included, 196 (82%) of whom after the starting of the collaboration project. At diagnosis 35/43(81.4%) vs 151/196 (77%) patients were smear sputum positive before and after the beginning of the project, respectively.

At 2 months follow up 23/35 (65.7%) patients in 2012 vs. 139/151 (92%) patients in 2013–2016 had negative control AFB stain (*p* = 0.249), 4/35 (11.4%) vs 12/151 (8%) patients remained AFB stain positive (*p* = 0.17), 7/35 (20%) vs 0/151 died before the 2 months follow up (*p* <  0.0001). TB treatment outcome was more frequently favourable after the beginning of cooperation 29/43 (67.4%) vs. 176/196 (89.8%) patients, (*p <*  0.0001). Patients’ mortality during treatment decreased from 8/43 (18.6%) in 2012 to 11/196 (5.6%) patients in the following years (*p* = 0.009).

**Conclusion:**

The implementation of diagnostic procedures, if integrated in a socio-economical intervention, impacts favourably on TB retention in care and treatment outcomes.

## Background

Ending the global tuberculosis (TB) epidemic by the year 2035 is one of the most ambitious goals of World Health Organization (WHO). The WHO strategy is based on three major pillars: improving TB prevention and care, supporting bold policies and enhancing research and innovation [1]. In 2016, 1.6 million people died from *Mycobacterium tuberculosis *(MTB) [2]. The prompt diagnosis of new cases is necessary to reduce TB burden, especially in low-income countries (LIC) where incidence is elevated and undiagnosed cases remain an oppressive issue [1, 2]. For example, in 2016, the total number of TB cases notified in the WHO African-region was above one million, accounting for an incidence of 254 cases every 100,000 inhabitants (range 227–284) [2]. In Senegal, where this project was conducted, the incidence of active TB seems to have increased since the last 15 years; in fact, in 2016 the incidence reached 140 (range 95–193) cases every 100,000 inhabitants (range 100–198) [2–4]. However, jeopardized case-finding and case-notification do not allow a precise estimation of the real TB burden in the whole country, which are merely based on case notifications and expert opinions [2, 4]. In addition, the gross domestic product of Senegal has been decreasing and more than 68% of the population in the Fatick region is currently living under the threshold of poverty, actively concurring to exacerbate the disease burden [5, 6]. The Senegalese “National Tuberculosis Programme” (NTP), in accordance with WHO guidelines, points to end the TB epidemic and suggests an appropriate treatment of all patients with clinical and/or microbiological and/or radiological signs of TB [4]. Since 2013 StopTB Italia Onlus sustains the Senegalese NTP, along with the economic aid of “Fondazione Monzino” and in collaboration with “Yungar per la pace” [7], a NGO that supports alphabetization of women in Senegal. The cooperation project aims to ameliorate TB treatment as well as to address the social/economic determinants of the illness. The policy consists in improving diagnostic capability with technological interventions, ameliorating educational programs for health care personnel, rising awareness among civil society and providing economical support for patients during treatment. The purpose of our study was to assess the results of an interventional cooperation project aimed at increasing illness awareness, ameliorate treatment outcome and providing patients’ economic support in a peripheral health care facility in Senegal where no data were previously available.

## Methods

### Location of the project

The present research was conducted at the Health Centre of Diofior (HCD), a tertiary level health care centre, serving the Fimela district in the Fatick region of Senegal. The HCD serves an area of 1115 km^2^ and an estimated population of 80,599 inhabitants (population density: 72 inhabitants per km^2^), mainly living in rural and sub-urban areas [8]. Currently, no precise epidemiological data about TB incidence or prevalence are available for the Fimela district.

### Pre-intervention diagnostic strategy

Until 2013, self-referred patients to the HCD, with presumptive symptoms of TB, according to the Senegalese NTP guidelines, underwent acid-fast bacillus stain (AFB) test and, if it resulted positive, HIV-screening test was proposed and anti-TB treatment was started. If the AFB test resulted negative, chest-X-ray in other health-care centres was proposed (but limited to the high price of the test and by the distance to other health-care centres). Patients’ self-referring to the HCD from the surrounding villages was restricted by limited awareness of the disease’s symptoms and by limited knowledge of treatment’s availability at the HCD.

### The StopTB project adopted at the HCD

The intervention consists of the following four main points that were gradually introduced since 2013. First, in order to increase awareness in the population, the TB suggestive symptoms according to WHO (productive cough for 10 days or more, fever, weight loss and night sweats) were enlisted in a diagnostic brochure and distributed in all villages of the Fimela district [2]. Due to high-illiteracy level in the area, idiomatic brochures were suitably created by StopTB Italia Onlus.

Second, 50 women (badieu’ngox) were selected, trained, supervised, annually retrained and paid to detect chronic cougher in the villages surrounding Diofior and to refer the patients to the HCD [7]. The badieu’ngox were selected upon their knowledge of the territory, reliability among the villagers as they are elected as social representative of the community and for their comprehension both of local dialects and French language. Moreover, they all had had trained in math and French writing by previous non-governative associations. Badieu’ngox were present before the arrival of StopTB Italia Onlus and they were already operating in social sectors of villages’ daily life (such as first aid, contributing to awareness of school services, exc.). Badieu’ngox main role is being a sentinel for patients with presumptive symptoms of TB and refer them to HCD.

Third, in February 2014, the laboratory equipment at the HCD was enriched with one four-module Xpert MTB/RIF system (Cepheid, Sunnydale, CA, USA), laboratory’s technicians were trained and supervised and retrained annually to its use and management.

Lastly, a digital chest-X-ray (CXR) mobile technology was donated in January 2016. An Italian radiology technician was delegated to teach how to use the digital mobile CXR and he performs annual retraining of the doctors at the HCD.

In addition, as a result of the cooperation project, in 2017 the city hall of Diofior spontaneously donated farmland to a selected number of patients who successfully completed the anti-TB treatment; the goal was to use the possible incomes from the cultivated farmland for the sustainment of the patients’ family and the maintenance of the project, in order to make it independent from external funds.

### The sequential diagnostic strategy

**Fig. 1:**
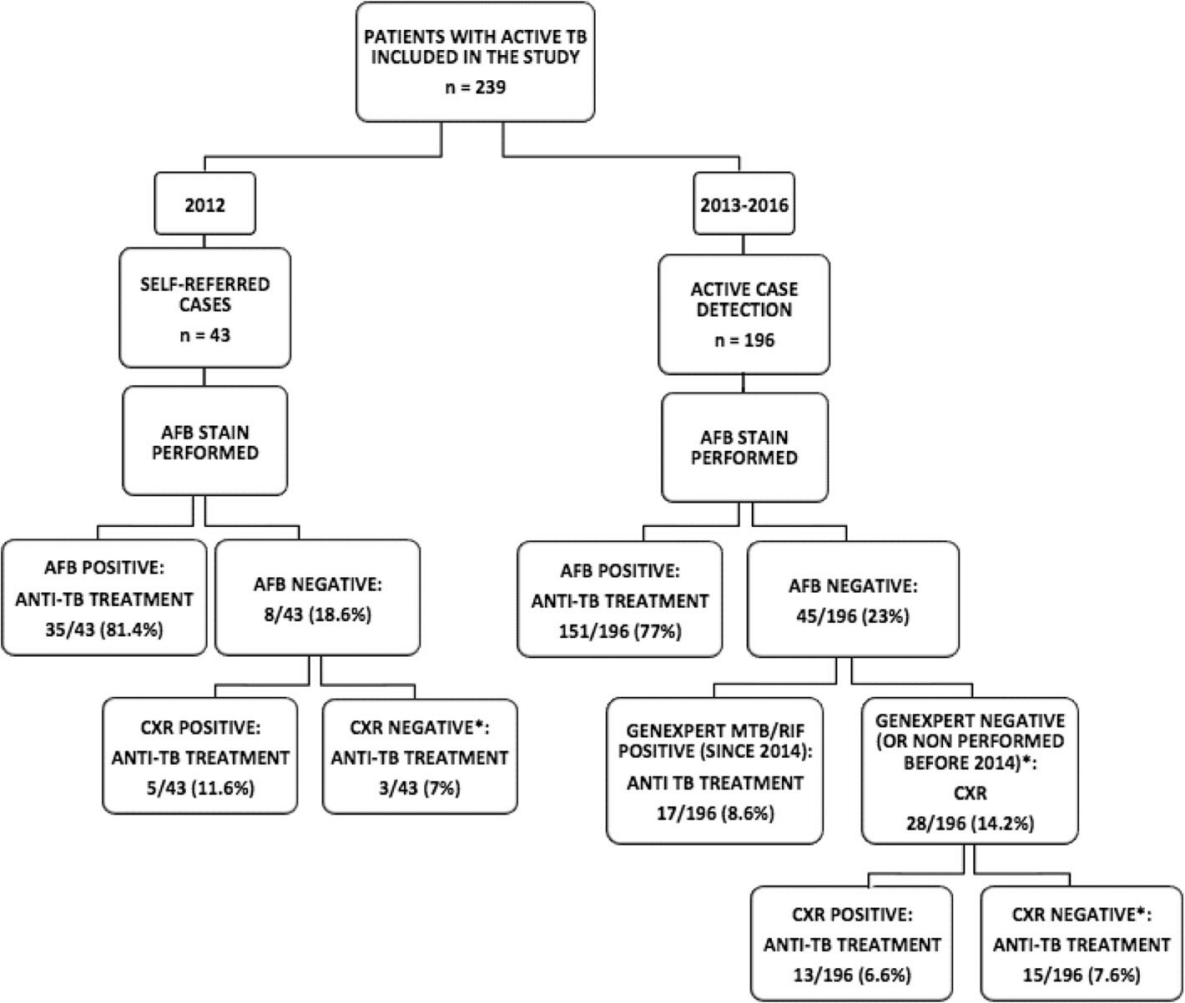
The sequential strategy adopted at the Health Care Centre of Diofior. TB = tuberculosis; AFB = acid-fast bacillus stain; CXR = chest-X-ray; * = high clinical suspicion cases with negative diagnostic surveys.

A sequential strategy for detection and treatment of TB new cases was progressively adopted at the HCD (Fig. 1).

All referred presumptive TB cases, firstly underwent AFB test, while culture test was not performed due to structural-laboratory limitations and due to the distance to the regional supranational laboratory (approximately 150 Km). Xpert MTB/RIF was used as a second-level diagnostic tool for negative AFB patients with high clinical suspicion of TB, according to NTP guidelines [4].

Due to the high-cost of films, chest-X-ray was relegated as third-level diagnostic tool for both negative AFB and Xpert MTB/RIF patients. Before the CXR donation, all chest-X-ray were performed in equipped centres outside the Fimela district and paid by the patients. HIV (1/2) testing was offered with opt-out strategy to all newly detected TB patients. All new cases were treated with isoniazid, rifampicin, pyrazinamide and ethambutol (HRZE) in fixed dose combination (FDC) (Rimstar®) for the 2 months intensive phase and with FDC-HR (Rifinah®) for the following 4 months of continuation phase; streptomycin (S) was added during the induction phase for failure and/or retreatment patients [2, 9].

All patients who started a TB treatment underwent follow up visits and AFB test at one, two, five and six months. Economic support for the patients, 20,000 Franc of the French Community of Africa (FCFA) (the equivalent of 35.8 US dollars) was offered to all patients who completed treatment in order to stimulate retention-in care [7]. Political commitment of the Senegalese government has been obtained to ensure the future sustainability of the activities after the end of the project.

### Data collection and study design

Data of active pulmonary (PTB) and extra-pulmonary tuberculosis (EPTB), from January 1^st^, 2012 to December 31^st^, 2016, were retrospectively and anonymously registered in an electronic database. Demographic characteristics (age, sex and geographical case-location) were collected in addition to microbiological parameters (AFB stain and molecular test) as well as radiological findings. Moreover, HIV status, TB retention in care and outcomes results were recorded. Treatment outcome, favourable or unfavourable, was defined according to 2016 WHO definitions [2, 9]. To assess the efficacy of the cooperation project, the outcomes of TB treatment were compared; patients were divided in two subgroups, whether diagnosed in year 2012 or thereafter. Chi square test or Fisher’s exact test were carried out to compare categorical variables. A logistical regression was used to compare the outcome of TB treatment before and after the project. Variables showing a *p* < 0.10 were included in a multivariable model if missing values were below 10%. Statistical analysis was performed using the Statistical Package for Social Science (SPSS version 20, IBM, USA). Legal agreement between StopTB Italia Onlus and the Health Care district of Diofior was settled before the beginning of the project. Local ethical committee approval was not required due the observational and retrospective design of the study.

## Results

Overall, 239 patients with TB were included, 196 (82%) of whom after the starting of StopTB Italia Onlus activities. Table 1 outlines the main features of the study population before and after the project.

**Table 1:** Baseline characteristics of patients from 2012 to 2016 (*n* = 239)

	Pre-intervention *n* = 43(%)	Post-intervention *n* = 196(%)	*p*
Sex, male	32 (72.1)	131 (66.8)	
Mean age (±SD)	31.7 (±15.5)	41.6 (±20.0)	
Performed HIV screening test at T0^1^	29 (67.5)	186 (94.9)	< 0.001
EPTB	4 (9.3)	13 (6.6)	0.37
AFB at T0 positive	35 (81.4)	151 (77)	
AFB at 2-months positive	4 (11.4)	12 (8)	0.17
AFB at 5-months positive	1 (4.2)	3 (2)	0.64
Favourable outcome	29 (67.4)	176 (89.8)	< 0.001
Mortality during treatment	8 (18.6%)	11 (5.6%)	0.009

*EPTB* extra pulmonary TB, *AFB* acid-fast bacillus stain, *CXR* chest-X-ray, *T0* performed at diagnosis

### Pre-intervention results

In 2012, 43 TB cases were detected. AFB at diagnosis was positive in 35/43(81.4%) patients. Of them, the AFB score resulted of 1+, 2+ and 3+ in 6(17.1%), 5 (14.3%) and in 24 (68.6%) cases, respectively. At 2 months follow up, 23 (65.7%) out of the 35 patients with sputum smear positive at diagnosis had AFB stain conversion, while 4 (11.4%) remained positive; 7 (20%) died before the 2 months follow up and 1 (2.9%) was not able to expectorate. At 5 months follow up, 23 (85.2%) previously sputum smear positive and still alive patients had AFB stain conversion, 1 (3.7%) was still positive, 3 (11.1%) were unable to expectorate and 1 (3.7%) patients had died before the scheduled visit. Due to high-clinical suspicion, but negative bacteriological or radiological findings, 3 patients were treated for TB with resolutions of symptoms.

The mean treatments duration for PTB new cases was 198.2 days (range: 74–244 days SD + − 76.1 days). Three (7%) patients were HIV-infected, but in 14 (32.5%) cases the HIV status was unknown.

In 2012, a favourable TB treatment outcome was reached in 29 (67.4%) cases; out of them 23 (53.4%) were treatment completed and 6 (14%) cured. Of patients who did not achieve a favourable TB treatment outcome, 8 (18.6%) died during the course of treatment, 3(7%) patients were lost to follow up and 3 (7%) transferred out.

### Post-intervention results

A total of 196 cases were detected. According to the WHO definitions, at the beginning of therapy, 170 (86.7%) of them were new patients, while 10(5.1%) were re-treatment, 6 (3.2%) were transferred from other centres, and 4 (2%) were failure. HIV-infection status was checked in 186 (94.9%) cases at the moment of TB diagnosis; 10 (5.4%)patients resulted TB/HIV co-infected. All co-infected TB/HIV patients were enrolled on antiretroviral therapy (ART) and counselled about the co-infection.

AFB at diagnosis was positive in 151 (77%)patients. The AFB score resulted of 1+, 2+, 3+ in 35 (23.2%), 33 (21.8%) and 82 (54.3%) patients, respectively; quantification of AFB stain was not available in one case. At 2 months follow up, 139 (92%) of the sputum smear positive at diagnosis had AFB stain conversion, while 12 (8%) remained positive.

At 5 months follow up, 143(94.7%) patients had control AFB stain conversion, while 3(2%) had positive control AFB stain, 3 (2%) lacked of expectoration and 2(1.3%) were transferred out.

Following diagnosis, all but one patients started treatment within 5 days. The mean treatment duration for new PTB cases was 193.7 days (range: 150–440 days, SD + − 28.5 days). Favourable treatment outcome was reached in 176 patients (89.8% of the cases) after the beginning of the cooperation, 34 (17.3% of the patients) were cured and 142(72.4%) completed the treatment course. Among TB patients who did not reach favourable treatment outcome: 10 (5.1%) patients died during the course of treatment, 4(2%) were transferred out, while 3 (1.5%) were treatment failure and other 3 (1.5%) were lost to follow up.

During the collaboration, patients underwent more frequently HIV screening (94% vs. 67.5%, *p* < 0.0001). Following the introduction of Xpert MTB/RIF, an overall of 17 samples were Xpert MTB/RIF positive and concomitantly AFB negative; no rpo-B mutations were detected.

The chest-X-ray led to an overall of 16 new TB diagnoses with concomitant negative AFB stain and negative molecular test. Out of those, six (37.5%) were made by the mobile radiology in the year 2016. Due to high-clinical suspicion, but negative bacteriological or radiological findings, 15 patients were treated for TB with resolutions of symptoms.

On the other hand, the two study periods did not differ in terms of proportion of microbiological confirmed cases at diagnosis *p* = 0.5336 95% CI (− 6.7075 to 18.4547) and AFB negative stain at 2 months (92.1% between 2013 and 2016 vs. 65.7% in 2012 *p* = 0.249) and 5 months of follow up (143 patients, 97.9% between 2013 and 2016 vs. 100.0% in 2012, *p* = 1.0).

**Fig. 2:**
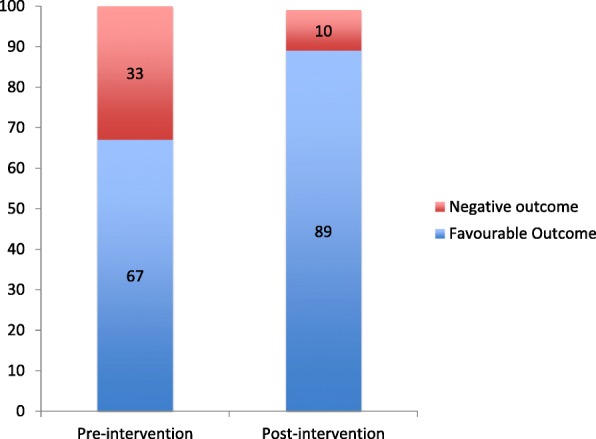
TB outcomes variations before and after StopTB Italia Onlus cooperation at the Health Care Centre of Diofior (percentage comparison). Negative outcome: dead, lost or transferred out during the follow up. Favorable outcome: cured or treatment-completed patients.

After the beginning of the project, TB treatment outcome was more frequently favourable (cured and treatment completed: 29 patients, 67.4% vs176 patients. 89.8%, *p* < 0.0001) (Fig. 2). The percentage of patients’ mortality during treatment decreased from 8 patients (18.6%) in 2012 to 11 patients (5.6%) in the following years (*p* = 0.009).

## Discussion

Case detection and retention in care of TB patients are challenging both in high and low income countries, especially in rural settings [10, 11]. Few studies reported on retention in care in LIC, where up to 70% of the patients are lost to follow up [12, 13]. This phenomenon is likely contributing to the amplification of drugs resistance, highlighting the need of new strategies for the implementation of TB retention in care and treatment [12, 14, 15].

The main finding of the present study is that the sequential interventions realized at HCD leaded to more favourable treatment outcomes and lower mortality rate compared to 2012 and compared to other LIC [16].While the TB treatment regimen was the same throughout the study period, cured and treatment completed rate markedly improved, as confirmed by high degree of smear negativity at the first microbiological follow up compared to other LIC settings [17, 18]. Additionally, AFB testing and AFB conversion rate at 2 and 5 months follow up did not differ in reason of the study period, further supporting improvement of retention in care.

No positive rpo-B samples were reported during the study period, confirming the low incidence compared to metropolitan areas as well as the low rate of treatment failure.

Another main finding of this study is that HIV testing was enhanced during the collaboration project, reaching 89% of TB patients and exceeding WHO estimates for Senegal [2]. Since 2013, HIV screening was proposed to every patient, and health care personnel received dedicated training, resulting in higher acceptance rate. The TB/HIV prevalence in the population study was concordant with co-infection prevalence estimated in 2011 by the NTP in Senegal [19, 20].

CXR examination, during the period 2013–2016, provided a presumptive diagnosis of 16 cases with concomitant negative AFB test and Xpert MTB/RIF, thus confirming the importance of CXR even if burdened by high cost (maintenance and films) in rural areas [21, 22]. In fact, while Xpert is free of charge, CXR has still to be paid by patients at HCD.

Finally, the cooperation project resulted in socio-economical support to all patients who completed anti-TB treatment, possibly contributing to community consciousness and decreased disease stigma [23, 24].

### Study limitations

The study was limited both by no data before 2012 for a 4-year comparison and by no data about the proportion of new cases versus previously treated in the 2012 subgroup. Moreover, before the beginning of the study, no precise epidemiological data were available for the Diofior area. Furthermore, the patients of the 2012 subgroup did not have the same diagnostic benefit and social support of the following subgroup. Additionally, while we assessed the overall results of the interventional cooperation project, we did not stratify the outcomes according with the single intervention procedures introduced.

## Conclusion

A systemic project based on TB awareness among the population, along with the implementation of diagnostic procedures in selected health care centres and a patient-centred approach, may favourably impacts on TB retention in care and treatment outcomes. Social and economic supports are fundamental pillars in the fights against TB, both to decrease risk factors and ameliorate patients’ conditions.

This comprehensive approach to control TB has been feasible and effective in a Senegalese rural area and may be exported to other similar settings.

## Abbreviations

AFB: Acid-Fast Bacillus stain
CXR: Chest X-Ray
EPTB: Extra-Pulmonary Tubercolosis
FCFA: Franc of the French Community of Africa
FDC: Fixed Dose Combination
FDC_HR: Fixed Dose Combination isoniazid, rifampicin
HCD: Health Centro of Diofior
HRZE: isoniazid, rifampicin, pyrazinamide and ethambutol
LIC: Low Income Countries
MTB: Mycobacterium Tuberculosis
MTB/RIF: Mycobacterium tuberculosis Rifampicin Resistant
NTP: National Tubercolosis Programme
PTB: Pulmonary Tubercolosis
SPSS: Social Package of Social Sciences
TB: tubercolosis
WHO: World Health Organization
